# Methodological issues in assessing change in dietary intake and appetite following gastric bypass surgery: A systematic review

**DOI:** 10.1111/obr.13202

**Published:** 2021-02-01

**Authors:** Tamsyn L. Redpath, M. Barbara E. Livingstone, Aoibheann A. Dunne, Adele Boyd, Carel W. le Roux, Alan C. Spector, Ruth K. Price

**Affiliations:** ^1^ Nutrition Innovation Centre for Food and Health Ulster University Coleraine UK; ^2^ Diabetes Complications Research Centre University College Dublin Dublin Ireland; ^3^ Department of Psychology and Program in Neuroscience Florida State University Tallahassee Florida USA

**Keywords:** appetite, dietary intake, gastric bypass, methodology

## Abstract

Gastric bypass surgery is an effective long‐term treatment for individuals with severe obesity. Changes in appetite, dietary intake, and food preferences have all been postulated to contribute to postoperative body weight regulation, however, findings are inconsistent. The aim of this systematic review was to evaluate the current literature on changes in dietary intake and appetite following gastric bypass surgery, in the context of the methodology used and the analysis, interpretation, and presentation of results. Four databases were systematically searched with terms related to “gastric bypass surgery,” “appetite,” and “dietary intake,” and 49 papers (*n* = 2384 patients after gastric bypass) were eligible for inclusion. The evidence indicated that only a reduction in overall energy intake and an increase in postprandial satiety are maintained beyond 6‐month post‐surgery, whereas relative macronutrient intake and premeal hunger remain unchanged. However, available data were limited by inconsistencies in the methods, analysis, presentation, and interpretation of results. In particular, there was a reliance on data collected by subjective methods with minimal acknowledgment of the limitations, such as misreporting of food intake. There is a need for further work employing objective measurement of appetite and dietary intake following gastric bypass surgery to determine how these mechanisms may contribute to weight regulation in the longer term.

**Abbreviations**
AUC: area under the curve
BMI: body mass index
ED: energy density
EI: energy intake
FFQ: food frequency questionnaires
gLMS: generalized labeled magnitude scale
GLP‐1: glucagon‐like peptide 1
h: hours
kg: kilograms
kJ: kilojoules
NS: nonsignificant
PYY: peptide YY
RCT: randomized control trials
T2DM: type 2 diabetes mellitus
VAS: Visual Analogue Scales

## 1 INTRODUCTION

Gastric bypass surgery is a successful established treatment for individuals with severe obesity (body mass index [BMI] ≥ 40 kg/m^2^ or a BMI > 35 kg/m^2^ with one or more associated comorbidities that would improve with weight loss), with weight loss superior to nonsurgical lifestyle interventions^1^ and maintained by the individual in the long term.^2^ In addition to weight loss, the surgery leads to improvements in comorbidities including type 2 diabetes mellitus (T2DM)^3^ and adverse cardiovascular events.^4^

The mechanisms behind weight loss and its maintenance following surgery are complex and not fully understood.^5^ Previously, weight loss was thought to be mediated primarily through stomach restriction and nutrient malabsorption, but these physical changes do not fully explain the successful long‐term body weight regulation. Postulated mechanisms contributing to the long‐term success of gastric bypass surgery include changes in circulating gut hormones,^6^ attenuated resting energy expenditure through the management and preservation of lean mass,^5^, ^7^ and changes in taste sensitivity thresholds,^8^, ^9^, ^10^, ^11^ which may affect food preferences and selection.

Postoperatively, patients typically report changes in appetite and dietary intake that may facilitate long‐standing weight maintenance. Overall daily energy intake (EI) is significantly reduced, with a recent meta‐analysis estimating an average postoperative reduction of 4.39 MJ/day,^12^ which is sustained in the longer term.^12^, ^13^, ^14^ It is unclear whether these changes in EI are facilitated through changes in macronutrient contribution to the diet, and reported findings regarding dietary macronutrient contribution to EI, at least in the short term, are equivocal.^15^ Animal studies have reported that rats avoid high‐fat stimuli^16^ and consume proportionally less fat in their diet following gastric bypass surgery.^16^, ^17^, ^18^, ^19^, ^20^, ^21^, ^22^ Although some studies have reported similar changes in humans^23^, ^24^ these proportional shifts in relative fat intake are not a consistent finding. Proposed mechanisms accounting for these possible shifts in macronutrient contribution to the diet of patients post‐gastric bypass surgery include changes in taste^25^ and diminished preference for palatable (high‐fat, high‐sugar) foods.^26^ Preserving or increasing dietary protein postoperatively may both increase satiety and protect against the loss of lean mass,^27^ whereas reducing dietary fat may reduce overall dietary energy density (ED) and thus EI.^24^

Changes in body fat set points in the longer term may also explain how reduced EI is maintained.^28^ The regulation of appetite occurs through physiological changes in the neuroendocrine system,^29^ as well as through sensory, cognitive, and physical processes that indicate feelings of hunger and satiety. Patients after gastric bypass surgery report improved postprandial satiety compared with control participants with obesity.^30^ The proposed mechanisms include changes in appetite‐regulating hormones, with postprandial appetite‐suppressing glucagon‐like peptide 1 (GLP‐1) and peptide YY (PYY) secretion increased postoperatively.^31^, ^32^

The assessment of appetite may be through objective (EI), subjective (e.g., Visual Analogue Scales [VASs]), or biochemical (e.g., circulating gut hormones) measures. The VASs are the most commonly utilized measure, which, while easy to administer, can be difficult to quantify and compare. Appetite sensations are subjective, with no two individuals experiencing these feelings in the same way,^33^ and so responses given on a VAS are not absolute values (e.g., a score of 40 mm on a hunger scale cannot be assumed to represent exactly half the hunger sensation of a score of 80 mm). Although new developments in scaling methodology have occurred over the last two decades such as the generalized labeled magnitude scale (gLMS),^34^, ^35^ which attempts to standardize for differential lifetime sensory experience, they are not yet widely adopted in bariatric surgery research with some exceptions.^11^ Nevertheless, such scales still depend on subjective verbal report and would be reinforced by complementary implementation of more direct objective measures.^15^

Studies often utilize subjective measures of both fasted and postprandial hunger and satiety in conjunction with objective measures of hormone levels to assess the impact of gastric bypass surgery. Patients have reported changes in fasted hunger as early as 2 days postoperatively, which correlate with changes in gut hormone profiles in the short term,^36^ but this is less consistent in the longer term.^31^, ^37^ Postprandial hunger and satiety are more variable and equivocal. The characterization of the effects of gastric bypass surgery on both fasted and postprandial appetite may lead to further insight into the underlying mechanisms of the success of bariatric surgery and help to predict which patients are likely to respond better to treatment.

Subjective measures (dietary recalls and dietary records) are also the method of choice when measuring free‐living dietary intake in bariatric populations. There is a tacit acceptance of the validity of EI data despite extensive evidence that these methods are highly susceptible to misreporting, with underreporting particularly frequent in participants with obesity.^38^ Although discrepant findings may be, in part, attributed to individual differences, it is likely that the current reliance on self‐reported measures of appetite and EI are contributing to inconsistent findings.

Previous reviews have also identified differences in the reporting of findings such as weight loss,^39^ patient‐reported outcomes,^40^ and clinical outcomes.^41^ Although there have been efforts to standardize the reporting of these outcomes,^42^ the inconsistencies prevent quantitative synthesis and make results difficult to compare. Thus, disparities in the methods, analysis, and presentation of outcomes relating to both appetite and dietary intake in bariatric patients may, at least partially, contribute to the confusion and lack of clarity observed in the current literature.

The aim of this review is to evaluate the current literature on changes in dietary intake and reported appetite following gastric bypass surgery, specifically in the context of the methodology used and the analysis, interpretation, and presentation of results from these studies.

## 2 METHODS

### 2.1 Search strategy

The Preferred Reporting Items for Systematic Reviews and Meta‐Analyses (PRISMA) guidelines^43^ informed the protocol for the systematic searching of the literature. This review was registered with the PROSPERO database (CRD42019126302).

Four databases (Embase, Medline, Scopus, and Cochrane) were systematically searched. Keyword searches were population based (“Gastric Bypass” OR “Roux en Y” OR “Bariatric surgery”) in combination with appetite (“Appetite” OR “Eating” OR “Hunger” OR “Satiation”) and dietary intake (“Energy Intake” OR “Dietary Fats” OR “Dietary Proteins” OR “Dietary Carbohydrates”) including their cognates and synonyms. Gray literature searches of conference outputs and reference lists were also completed.

### 2.2 Inclusion/exclusion criteria

Included papers were peer‐reviewed observational or intervention studies that were published in English from January 1990 to August 2017. Studies had to present quantitative data on human subjects who had undergone gastric bypass surgery, and all studies were required to include a measure of changes in dietary intake and/or reported appetite. Full inclusion and exclusion criteria are presented in Table 1.

**TABLE 1:** The inclusion and exclusion criteria applied in the systematic literature review

Inclusion criteria	Exclusion criteria
▪ Published in the English language between January 1, 1990, and August 31, 2017 ▪ At least one group within the study has undergone gastric bypass surgery ▪ Adults and/or adolescents. ▪ Include a comparative measurement (e.g., pre‐ to post‐) ▪ Quantitative studies only ▪ Intervention/observational studies ▪ Original articles	▪ Not undergoing gastric bypass surgery, including other bariatric surgeries later converted to gastric bypass ▪ Studies conducted in animals, *in vitro*, or *ex vivo* ▪ Qualitative studies ▪ Studies with no comparator group to measure change

### 2.3 Data selection and extraction

A single reviewer (TR) completed an initial screen of all titles and abstracts retrieved from the systematic searches. A second reviewer (AD) then completed an independent screen to ensure no eligible papers were excluded. These two reviewers independently assessed the selected papers for full text screening and determined which should be included for review. A third reviewer (a member of the authorship team) was available to adjudicate any differences in opinion between the two reviewers, of which there were none.

**FIGURE 1:**
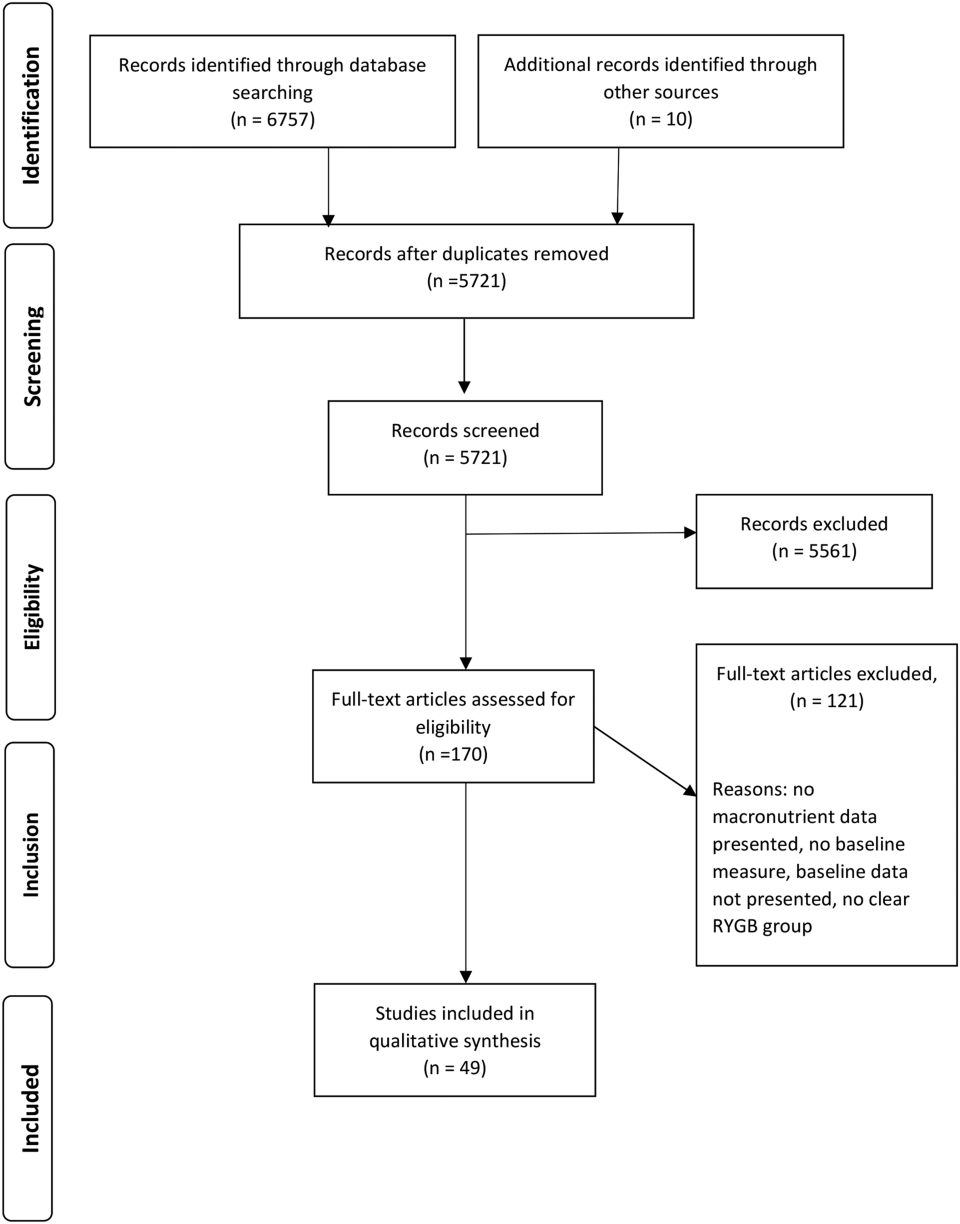
An overview of the literature searches and inclusion process for this systematic review (adapted from PRISMA guidelines [41.PRISMA, 2009])

The electronic database searches initially returned 6757 studies. After duplicates were removed, there were 5711 papers that underwent title and abstract screening. Gray literature searches returned an additional 10 papers to be included. Following this initial screen, 170 titles were left for full text review. Forty‐nine papers were considered suitable, having met the inclusion criteria, and data were extracted for review. After completing the full screen, retained papers were assessed using the Critical Appraisal Skills Programme.^44^ Figure 1 provides an overview of the literature searches.

Primary outcome data extracted for review from included papers were changes in fasted hunger, postprandial appetite, EI, and dietary composition following gastric bypass surgery. Secondary outcomes extracted (where available) were changes in circulating gut hormones, food selection, dietary ED, and micronutrient intake. Data extracted on methodology included measures used, sample size (and %follow‐up), study duration, length of fast prior to appointment, composition of preload, influence of misreporting, and subject selection.

## 3 RESULTS

### 3.1 Study characteristics

Following screening, 49 studies were eligible for inclusion. Most studies were of an observational design, with only two randomized controlled trials (RCT) (4%), two non‐RCT (4%), and one cross‐sectional trial (2%). Twenty‐seven (54%) of the studies were conducted in Europe, 14 (28%) in North America, and nine (18%) in South America.

In total, 2384 participants were included, with sample size ranging from 5 to 294. Participant completion rate varied from 26% to 100%. Approximately two thirds of participants were female (*n* = 1,657), with 10 studies measuring changes in females only. Recruitment for each study was from one site (e.g., one hospital), except for one study that recruited across two sites (Norway/Sweden).^45^ No studies measured changes in participants who had undergone one‐anastomosis gastric bypass and only one assessed postsurgical changes in adolescents. Only four papers specified that medications that may affect intake/metabolism/weight loss were an exclusion criterion or that data were collected on medications, with none presenting results on medication usage. The duration of follow‐up ranged from 1‐month to 8‐year post‐surgery.

### 3.2 Daily EI

Postoperative changes in EI were reported in 30 (61%) studies. Change was mostly expressed as total EI/day, although it was also reported as EI adjusted for BMI (*n* = 1) and EI/kg body weight (*n* = 2). All studies that presented change in EI/day reported a reduction in EI up to 8‐year post‐surgery. When presented in terms of body size, findings were more inconsistent. In adolescents, changes in EI (adjusted for BMI) decreased up until 3‐month post‐surgery, although changes were no longer significant by 1‐year post‐surgery.^46^ In adults, two studies observed a decrease in EI/kg up to 6‐month post‐surgery.^47^, ^48^ However, at 1‐year post‐surgery, they diverged, with one study observing a consistent reduction in EI/kg,^47^ whereas the other reported that changes were no longer significant at 1‐year post‐surgery, with EI/kg significantly increasing at 2 years postoperatively.^48^

All studies that measured dietary intake used subjective self‐reported measures, either dietary records (56%) (Table 2) or dietary recalls (44%) (Table 3). The duration of dietary recording varied from 3 to 7 days, and dietary recalls varied in format from recalls (*n* = 8), food frequency questionnaires (FFQ) (*n* = 3), and specially adapted questionnaires for bariatric populations (*n* = 3). Ten (33%) papers noted that a dietitian had either administered or reviewed the dietary intake measurements, whereas three specified that a research nutritionist obtained dietary intake data (Tables 2 and 3).

**TABLE 2:** Summary of methods and outcomes of studies measuring changes in energy and macronutrient intake in patients after gastric bypass from pre‐ to post‐surgery as assessed by dietary records, organized by follow‐up duration

Author (year)	Location	*N* (baseline)	*N* (follow‐up)	% follow‐up	Postoperative follow‐up time	Diet record duration	Method notes	Energy intake	Macronutrient intake change		
									Protein (%EI)	Fat (%EI)	CHO (%EI)
Jeffreys et al. (2012)^46^	USA	27	16	59	2 weeks	3 days	Dietary record reviewed by dietitian	↓	↑	↓	NS
Miller et al. (2014)^52^	USA	26	17	65	3 weeks	4 days	Research nutritionist instructed how to complete	↓	↓	↓	NS
Aron‐Winewsky et al. (2016)^49^	France	14	14	100	1 month	3 days	2 weekdays/1 weekend	↓	‐	↑	↓
Trostler et al. (1995)^90^	Israel	19	19	100	1 month	7 days	Verified w/24‐h recall	↓	↓	↓	↑
Trostler et al. (1995)^90^	Israel	19	19	100	2 months	7 days	Verified w/24‐h recall	↓	↓	↓	↑
Aron‐Winewsky et al. (2016)^49^	France	14	14	100	3 months	3 days	2 weekdays/1 weekend	↓	‐	NS	NS
Bobbioni‐Harsch et al. (2002)^50^	Switzerland	50	50	100	3 months	3 days	Verified by interview with a dietitian	↓	↑	NS	↓
Jeffreys et al. (2012)^46^	USA	27	11	41	3 months	3 days	Dietary record reviewed by dietitian	↓	↑	NS	↑
Miller et al. (2014)^52^	USA	26	17	65	3 months	4 days	Research nutritionist instructed how to complete	↓	↓	NS	↓
Trostler et al. (1995)^90^	Israel	19	19	100	3 months	7 days	Verified w/24‐h recall	↓	↓	↓	↑
Verger et al. (2016)^59^	France	22	22	100	3 months	3 days	Web‐based dietary record (2 weekdays/1 weekend)	↓	‐	NS	NS
Moize et al. (2013)^91^	Spain	25	25	100	4 months	3 days	‐	↓	↓	↓	↑
Trostler et al. (1995)^90^	Israel	19	19	100	4 months	7 days	Verified w/24‐h recall	↓	↓	↓	↑
Trostler et al. (1995)^90^	Israel	19	19	100	5 months	7 days	Verified w/24‐h recall	↓	↓	↓	↑
Bobbioni‐Harsch et al. (2002)^50^	Switzerland	50	50	100	6 months	3 days	Verified by interview with a dietitian	↓	↑	NS	↓
Coupaye et al. (2014)^60^	France	43	43	100	6 months	4 days	Verified by interview with a dietitian	↓	NS	NS	NS
Miller et al. (2014)^53^	USA	26	17	65	6 months	4 days	Research nutritionist instructed how to complete	↓	↓	NS	↓
Moize et al. (2013)^92^	Spain	294	294	100	6 months	3 days	Instructions on completion given by dietitian	↓	↑	NS	NS
Trostler et al. (1995)^90^	Israel	19	17	89	6 months	7 days	Verified w/24‐h recall	↓	↓	↓	↑
Trostler et al. (1995)^90^	Israel	19	12	63	9 months	7 days	Verified w/24‐h recall	↓	NS	NS	NS
Bobbioni‐Harsch et al. (2002)^50^	Switzerland	50	50	100	12 months	3 days	Verified by interview with a dietitian	↓	↑	NS	↓
Coupaye et al. (2014)^60^	France	43	30	70	12 months	4 days	Verified by interview with a dietitian	↓	NS	↓	↑
Jefferys et al. (2012)^46^	USA	27	9	33.3	12 months	3 days	Dietary record reviewed by dietitian	NS	↑	NS	↑
Miller et al. (2014)^24^	USA	26	17	65	12 months	4 days	Research nutritionist instructed how to complete	↓	↓	NS	NS
Moize et al. (2013)^92^	Spain	294	294	100	12 months	3 days	Dietitian instructed how to complete	↓	↑	NS	NS
Moize et al. (2013)^91^	Spain	25	25	100	12 months	3 days	‐	↓	↓	↓	↑
Trostler et al. (1995)^90^	Israel	19	12	63	12 months	7 days	Verified w/24‐h recall	↓	NS	NS	NS
Verger et al. (2016)^59^	France	22	14	63	12 months	3 days	Web‐based dietary record (2 weekdays/1 weekend)	↓	‐	NS	NS
Trostler et al. (1995)^90^	Israel	19	NA	NA	15 months	7 days	Verified w/24‐h recall	↓	NS	NS	NS
Trostler et al. (1995)^90^	Israel	19	11	58	18 months	7 days	Verified w/24‐h recall	↓	NS	NS	NS
Moize et al. (2013)^92^	Spain	294	259	88	24 months	3 days	‐	↓	NS	NS	NS
Moize et al. (2013)^92^	Spain	294	203	69	48 months	3 days	Dietitian instructed how to complete	↓	NS	NS	NS
Moize et al. (2013)^92^	Spain	294	138	47	60 months	3 days	Dietitian instructed how to complete	↓	NS	NS	NS
Kruseman et al. (2010)^14^	Switzerland	141	80	59	8 years	4 days	Dietitian instructed how to complete and reviewed dietary records	↓	‐	↓	NS

*Note*: All data measured as change from baseline following gastric bypass surgery. Data organized by follow‐up duration from shortest time postoperatively.

Abbreviations: EI, energy intake; FFQ, food frequency questionnaire; NS, no significant change from preoperative values; ↓, significantly lower than preoperative values; ↑, significantly higher than preoperative values; −, data not reported.

**TABLE 3:** Summary of methods and outcomes of studies measuring changes in energy and macronutrient intake in patients after gastric bypass from pre‐ to post‐surgery as assessed by dietary recall methods, organized by follow‐up duration

Author (year)	Location	*N* (baseline)	*N* (follow‐up)	% follow‐up	Postoperative follow‐up time	Measure	Method notes	Energy intake	Macronutrient intake change		
									Protein (%EI)	Fat (%EI)	CHO (%EI)
Bavaresco et al. (2010)^53^	Brazil	48	48	100	1 month	24‐h recall	‐	↓	↓	↓	↑
Laurenius et al. (2013)^24^	Sweden	43	42	98	6 weeks	Dietary questionnaire	Self‐administered	↓	‐	↓	‐
Anderson et al. (2007)^47^	USA	50	50	100	3 months	Dietary logs	Reviewed by dietitian	↓	↑	‐	‐
Bavaresco et al. (2010)^53^	Brazil	48	48	100	3 months	24‐h recall	‐	↓	NS	↓	↑
Lima et al. (2014)^58^	Brazil	29	‐	‐	3 months	24‐h recall	‐	↓	NS	NS	NS
Sarwer et al. (2008)^54^	USA	200	198	99	20 weeks	Block 98 FFQ	‐	↓	NS	NS	NS
Bavaresco et al. (2010)^53^	Brazil	48	48	100	6 months	24‐h recall	‐	↓	NS	NS	NS
Brolin et al. (1994)^23^	USA	108	108	100	6 months	24‐h recall	Obtained by nutritionist	↓	NS	↓	NS
Kenler et al. (1990)^55^	USA	51	48	94	6 months	24‐h recall	Obtained by nutritionist	↓	↑	NS	NS
Lima et al. (2014)^58^	Brazil	29	‐	‐	6 months	24‐h recall	‐	↓	NS	NS	NS
Molin‐Netto et al. (2017)^93^	Brazil	41	41	100	6 months	FFQ	Obtained through interview with dietitian	↓	NS	↓	↑
Bavaresco et al. (2010)^53^	Brazil	48	48	100	8 months	24‐h recall	‐	↓	NS	NS	NS
Sarwer et al. (2008)^54^	USA	200	147	74	40 weeks	Block 98 FFQ	‐	↓	NS	NS	NS
Anderson et al. (2007)^47^	USA	50	50	100	12 months	Dietary logs	Reviewed by dietitian	↓	↑	‐	‐
Bavaresco et al. (2010)^53^	Brazil	48	48	100	12 months	24‐h recall	‐	↓	NS	NS	NS
Brolin et al. (1994)^23^	USA	108	108	100	12 months	24‐h recall	Obtained by nutritionist	↓	NS	NS	NS
Johnson et al. (2013)^51^	Norway	76	72	95	12 months	FFQ	‐	↓	NS	NS	NS
Kenler et al. (1990)^55^	USA	51	43	84	12 months	24‐h recall	Obtained by nutritionist	↓	↑	NS	NS
Laurenius et al. (2013)^24^	Sweden	43	41	95	12 months	Dietary questionnaire	Self‐administered	↓	‐	↓	‐
Lima et al. (2014)^58^	Brazil	29	18	62	12 months	24‐h recall	‐	↓	NS	NS	NS
Sarwer et al. (2008)^54^	USA	200	92	46	66 weeks	Block 98 FFQ	‐	↓	NS	NS	NS
Brolin et al. (1994)^23^	USA	108	108	100	18 months	24‐h recall	Obtained by nutritionist	↓	NS	NS	NS
Kenler et al. (1990)^55^	USA	51	45	88	18 months	24‐h recall	Obtained by nutritionist	↓	NS	NS	NS
Brolin et al. (1994)^23^	USA	108	108	100	24 months	24‐h recall	Obtained by nutritionist	↓	NS	NS	NS
Kenler et al. (1990)^55^	USA	51	44	86	24 months	24‐h recall	Obtained by nutritionist	↓	NS	NS	NS
Laurenius et al. (2013)^24^	Sweden	43	41	95	24 months	Dietary questionnaire	Self‐administered	â	‐	↓	‐
Sarwer et al. (2008)^54^	USA	200	112	56	92 weeks	Block 98 FFQ	‐	↓	NS	NS	NS
Brolin et al. (1994)^23^	USA	108	108	100	36 months	24‐h recall	Obtained by nutritionist	↓	NS	↓	↑

*Note*: All data measured as change from baseline following gastric bypass surgery. Data organized by follow‐up duration from shortest time postoperatively.

Abbreviations: EI, energy intake; FFQ, food frequency questionnaire; NS, no significant change from preoperative values; ↓, significantly lower than preoperative values; ↑, significantly higher than postoperative values; −, data not reported.

When assessing self‐reported dietary intake, only three studies^24^, ^49^, ^50^ evaluated the possibility of misreporting but differed in the method of calculation and in the interpretation of the outcomes. Only Laurenius et al.^24^ adjusted data based on misreporting calculations, removing two participants they deemed were overreporting based on EI (>251 kJ/kg). Several studies (Tables 2 and 3) employed dietary interviews or recalls in relative validation of dietary records, or vice versa.

Twelve (40%) studies specified methods that enabled participants to more accurately report their portion sizes. Methods varied between household measures (25%), photographs (25%), artificial food models (17%), retail pack sizes (8%), or a combination (25%). None of the included studies specifically excluded fluid intake, and only Laurenius et al.^24^ were specific about not measuring water intake in their calculation of ED (kJ/g).

### 3.3 Macronutrient intake

Twenty‐nine studies (59%) measured changes in macronutrient intake in 1922 (range: 8–294) patients after gastric bypass. Data were presented either in relative (%EI) (*n* = 19, 66%), absolute (g/day) (*n* = 9, 31%), or relative to body weight (g/kg/day) (*n* = 1, 3%) terms.

**FIGURE 2:**
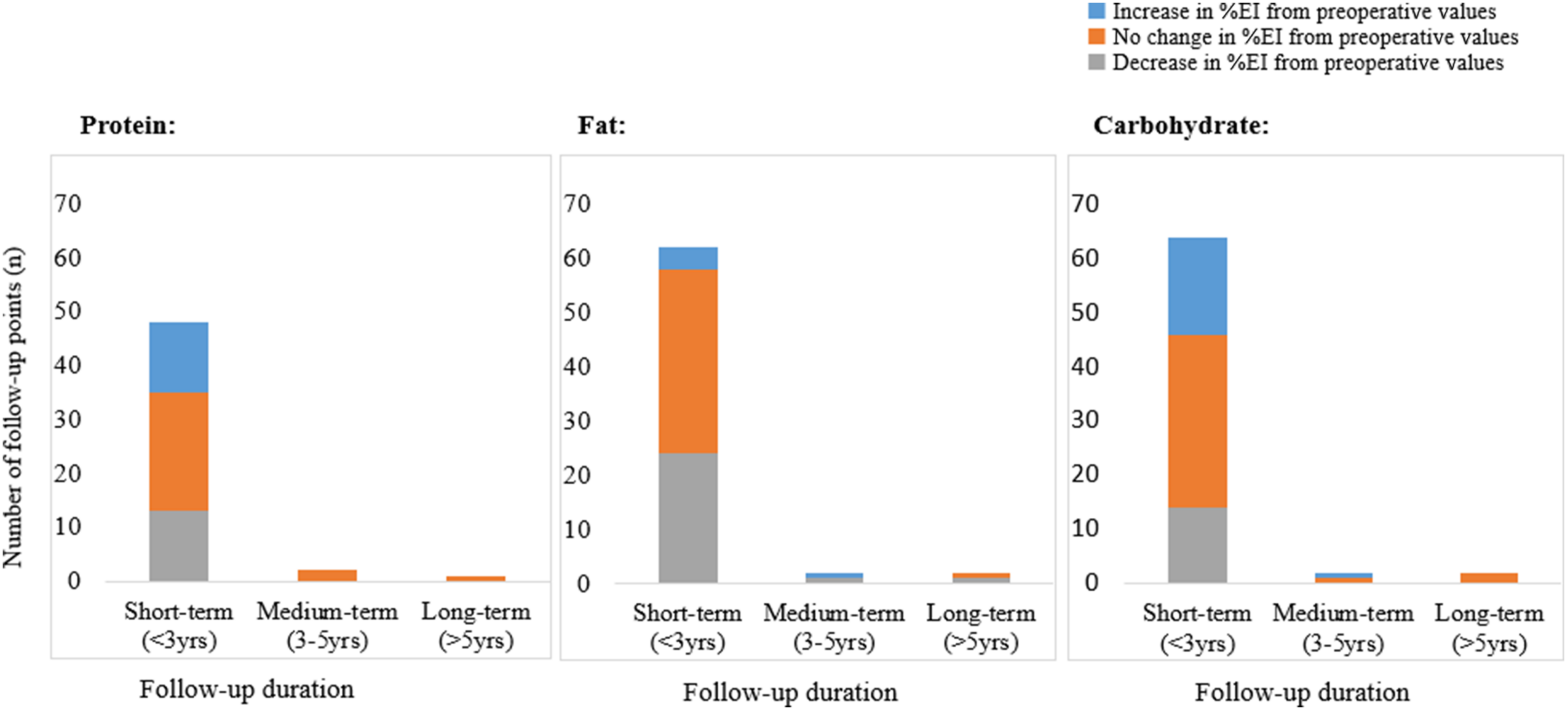
Findings (increase/decrease/no change) from published literature measuring % change in relative macronutrient intake in patients from pre‐ to post‐ surgery, organized by follow‐up duration. Follow‐up durations defined by Brethauer et al. (40‐2015)^42^. Data sourced from papers summarized in Table 2

Approximately three‐quarters of the studies that presented change in macronutrient intake as g/day or g/kg/day reported a decrease in intake in line with the reported decrease in EI. When relative macronutrient intake was evaluated (%EI), it was subdivided by duration of participant follow‐up defined as short (<3 years, 94%), medium (3–5 years, 3%), and long term (>5 years, 3%).^42^ When defined in this way, changes in relative macronutrient intake were inconsistent in the short term, whereas the majority of studies reported no change in the medium and long term (Figure 2). However, the paucity of studies with medium‐ and long‐term follow‐up preclude definitive conclusions.

**FIGURE 3:**
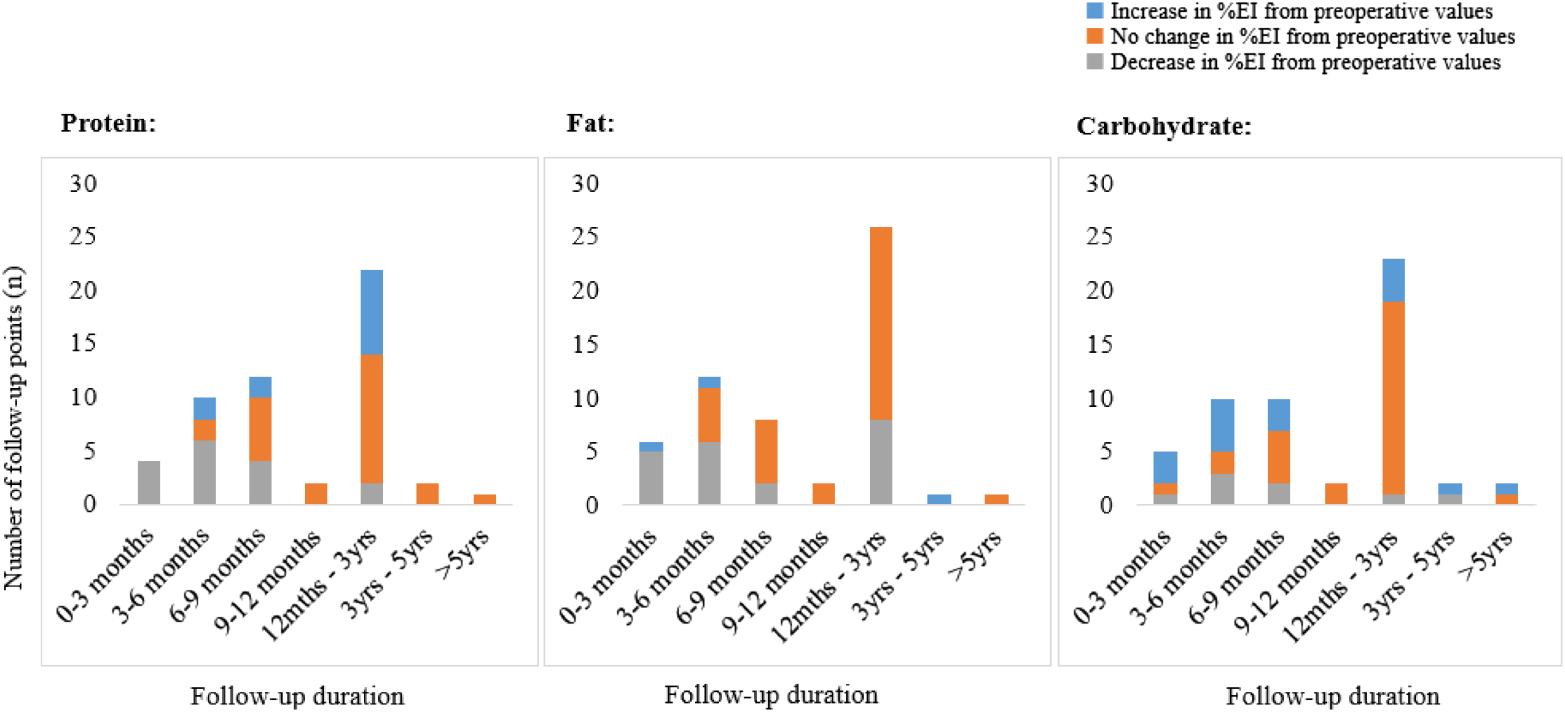
Findings (increase/decrease/no change) from published literature measuring % change in relative macronutrient intake in patients from pre‐ to post‐ surgery, organized by follow‐up duration. Data organized by 3‐month intervals up to 1‐year post‐surgery and sourced from papers summarized in Table 2

Given the disproportionate representation of short‐term follow‐up, data were further subdivided into 3‐month intervals up to 1‐year post‐surgery (0–3 months, 3–6 months, 6–9 months, 9–12 months; Figure 3) for a more detailed assessment of changes in intake patterns. From 0 to 3 months, most studies (80% to 86%) reported a decrease in protein and fat intake, with a reciprocal but nonsignificant increase in carbohydrate intake. By 6 months, most studies (70% to 75%) observed no change in the pattern of macronutrient intake, and this pattern was maintained at 9–12 months and 1–3 years' post‐surgery.

Only three studies measured sugar independently of total carbohydrate, but sugar was variously defined as sucrose,^50^ added sugar,^51^ or dietary sugar.^52^ When presented as g/day, sugar intake decreased.^52^ However, when presented as %EI, findings were inconsistent and reported as either a decrease from pre‐surgery intake^50^ or no different^51^ at 1‐year post‐surgery.

Dietary fiber intake (g/day) was assessed in three studies,^51^, ^52^, ^53^ and all reported a significant decrease in dietary fiber from pre‐ to 1‐year post‐surgery.

Dietary ED was measured in only one study.^24^ Intake of high‐ED foods (>16.7 kJ/kg) decreased at 6 weeks postoperatively, with a reciprocal increase in very‐low‐ED foods (<2.5 kJ/g). Only the increase in consumption of very‐low‐ED foods was maintained at 1‐year post‐surgery, but changes were no longer evident at 2 years. This was not reflected in overall dietary ED (kJ/g), which remained significantly decreased at 2‐year post‐surgery.

### 3.4 Food selection

Alongside the assessment of EI and macronutrient intake, food selection was also assessed in several studies. Most frequently examined were intakes of sweet foods, categorized as “desserts”^37^, ^54^ or as “sweets/sodas” and “milk/ice cream,”^23^, ^55^ with a decrease in the consumption of these foods observed in most studies (*n* = 3) up to 2‐year post‐surgery. When followed up at 36 months, Brolin et al.^23^ reported that the decrease in intake was no longer evident. This initial decrease was also observed by Miller et al.^52^ when assessing intake of “sweets” and “sweetened beverages,” but the return to preoperative intake levels was observed earlier (3 months postoperatively).

Different food groups were also assessed, but because of the lack of homogeneity between studies on how foods were grouped and defined, it is difficult to draw meaningful conclusions from the data. For example, fruit/vegetable intakes were variously categorized as fruit and vegetables, fruits, or vegetables or as “healthy” foods.

### 3.5 Micronutrient intake

Eight studies measured micronutrient intake and status. Most frequently measured were iron,^52^, ^56^, ^57^ calcium,^46^, ^56^, ^57^ and potassium.^52^, ^58^ All studies reported a decrease in the intake of these micronutrients in line with reported decreases in EI.

When micronutrient status was assessed, rates of deficiency did not change post‐surgery with several studies reporting that supplementation stabilized nutrient status following surgery.^49^, ^59^, ^60^

### 3.6 Subjective assessment of appetite

Nineteen studies measured subjective appetite in 362 (range: 5–76) patients. Studies measuring subjective appetite were smaller and shorter in duration compared with studies evaluating dietary intake. In addition, all patient follow‐up was in the short term only, and the outcomes were considered at 3‐month intervals.

#### 3.6.1 Premeal hunger

Twelve studies used VAS to assess premeal hunger (Table 4). Antecedent diet was not recorded by any study, although some specified that participants should abstain from alcohol consumption in the 24 h prior to measurement. Most studies (*n* = 15, 75%) stipulated a 12 h/overnight fast, although studies that employed objective assessments in conjunction with VAS implemented shorter duration of fasting (less than 5 h).^37^, ^61^ One study^62^ did not specify the duration of fast, whereas another did not stipulate a period of fasting prior to subjective assessment of premeal appetite.^63^

**TABLE 4:** Summary of methods and outcomes of papers measuring subjective fasted appetite changes in patients after gastric bypass from pre‐ to post‐surgery, organized by follow‐up duration

Author (year)	Study type	Location	*N* (baseline)	*N* (follow‐up)	% follow‐up	Postoperative follow‐up time	Measure	Duration of fast	Premeal hunger
le Roux et al. (2007)^36^	Observational	UK	16	16	100	2 days	VAS (100 mm)	12 h	↓
Falken et al. (2011)^31^	Observational	Sweden	12	12	100	3 days	VAS (100 mm)	Overnight	NS
le Roux et al. (2007)^36^	Observational	UK	16	16	100	4 days	VAS (100 mm)	12 h	↓
le Roux et al. (2007)^36^	Observational	UK	16	16	100	7 days	VAS (100 mm)	12 h	↓
Shankar et al. (2017)^62^	Observational	USA	17	17	100	2 weeks	VAS (100 mm)	‐	↓
Karakamos et al. (2008)^94^	Randomized controlled trial	Greece	16	16	100	1 month	VAS (100 mm)	Overnight	NS
Ochner et al. (2011)^64^	Observational	USA	5	5	100	1 month	VAS (100 mm)	Overnight	↓
Shankar et al. (2017)^62^	Observational	USA	17	17	100	4 weeks	VAS (100 mm)	‐	↓
le Roux et al. (2007)^36^	Observational	UK	16	16	100	42 days	VAS (100 mm)	12 h	↓
Laurenius et al. (2012)^37^	Observational	Sweden	43	42	98	6 weeks	VAS (100 mm)	5 h	NS
Moringo et al. (2006)^95^	Observational	Spain	9	9	100	6 weeks	VAS (100 mm)	Overnight	↓
Yousseif et al. (2014)^96^	Observational	UK	10	10	100	6 weeks	VAS (100 mm)	Overnight	↓
Schmidt et al. (2016)^30^	Observational	Denmark	17	16	94	7 weeks	VAS (100 mm)	Overnight	NS
Falken et al. (2011)^31^	Observational	Sweden	12	12	100	2 months	VAS (100 mm)	Overnight	NS
Morrow et al. (2008)^97^	Observational	USA	24	‐	‐	2 months	VAS (100 mm)	Overnight	↓
Valderas et al. (2010)^98^	Non‐randomized controlled trial	Chile	8	8	00	2 months	VAS (100 mm)	Overnight	NS
Schmidt et al. (2016)^30^	Observational	Denmark	17	47	82	11 weeks	VAS (100 mm)	Overnight	NS
Karakamos et al. (2008)^94^	Randomized controlled trial	Greece	16	12	100	3 months	VAS (100 mm)	Overnight	NS
Svane et al. (2016)^61^	Observational	Denmark	9	9	100	3 months	VAS (100 mm)	4 h	NS
Yousseif et al. (2014)^96^	Observational	UK	10	10	100	12 weeks	VAS (100 mm)	12 h	↓
Morrow et al. (2008)^97^	Observational	USA	24	‐	‐	5 months	VAS (100 mm)	Overnight	↓
Karakamos et al. (2008)^94^	Randomized controlled trial	Greece	16	16	100	6 months	VAS (100 mm)	Overnight	NS
Falken et al. (2011)^31^	Observational	Sweden	12	12	100	12 months	VAS (100 mm)	Overnight	NS
Karakamos et al. (2008)^94^	Randomized controlled trial	Greece	16	16	100	12 months	VAS (100 mm)	Overnight	NS
Laurenius et al. (2012)^37^	Observational	Sweden	43	41	95	12 months	VAS (100 mm)	5 h	NS
Schmidt et al. (2016)^30^	Observational	Denmark	30	21	70	78 weeks	VAS (100 mm)	Overnight	NS
Laurenius et al. (2012)^37^	Observational	Sweden	43	42	98	24 months	VAS (100 mm)	5 h	NS

*Note*: All data measured as change from baseline following gastric bypass surgery. Data organized by follow‐up duration from shortest time postoperatively.

Abbreviations: NS, no significant change from preoperative values; VAS, Visual Analogue Scale; ↓, significantly lower than preoperative values.

No study observed an increase in premeal hunger. In the early postoperative stages, over half reported a decrease in premeal hunger, but from 6 months onwards, studies were reporting no change in premeal hunger. In the absence of long‐term follow‐up studies, the significance of these findings is unclear.

#### 3.6.2 Postprandial appetite

Eighteen studies measured postprandial appetite sensations using assessments of satiety, hunger, fullness, and/or desire to eat either as individual measurements or in variable combination (Table 5). Postprandial satiety was the most frequently assessed sensation (*n* = 14, 77%). Up until 3‐month post‐surgery, just over two thirds of studies reported an increase in satiety, which were mostly maintained at 1–3 years' post‐surgery. Again, a lack of long‐term follow up makes the significance of these findings difficult to interpret.

**TABLE 5:** Summary of methods and outcomes of papers measuring subjective postprandial appetite changes in patients after gastric bypass from pre‐ to post‐surgery, organized by follow‐up duration

Author (year)	Study type	Location	*N* (baseline)	*N* (follow‐up)	*N* (%)	Postoperative follow‐up time	Measure (time)	Type (kJ)	Load			Postprandial hunger	Postprandial satiety	Postprandial fullness	Postprandial desire to eat
									Pro (%EI)	Fat (%EI)	CHO (%EI)				
le Roux et al. (2007)^36^	Observational	UK	16	16	100	2 days	VAS (100 mm) 180 min	Mixed meal (1674 kJ)	‐	‐	‐	‐	‐	↑	‐
Bryant et al. (2013)^99^	Observational	UK	12	12	100	3 days	VAS (100 mm) 180 min	200 ml liquid (1255 kJ)	11%	0%	89%	NS	NS	NS	NS
Falken et al. (2011)^31^	Observational	Sweden	12	12	100	3 days	VAS (100 mm) 180 min	200 ml liquid (1255 kJ)	11%	0%	89%	↓	‐	↑	‐
le Roux et al. (2007)^36^	Observational	UK	16	16	100	4 days	VAS (100 mm) 180 min	Mixed meal (1674 kJ)	‐	‐	‐	‐	‐	↑	‐
le Roux et al. (2007)^36^	Observational	UK	16	16	100	7 days	VAS (100 mm) 180 min	Mixed meal (1674 kJ)	‐	‐	‐	‐	‐	↑	‐
Karakamos et al. (2008)^94^	Randomized controlled trial	Greece	16	16	100	1 month	VAS (100 mm) 120 min	Mixed Meal (1757 kJ)	16%	29%	55%	NS	NS	‐	‐
Ochner et al. (2012)^100^	Observational	USA	5	5	100	1 month	VAS (100 mm)‐	250 ml liquid (1035 kJ)	‐	‐	‐	NS	‐	NS	‐
Ochner et al. (2011)^64^	Observational	USA	10	10	100	1 month	VAS (100 mm)‐	250 ml liquid (1035 kJ)	‐	‐	‐	‐	‐	‐	↓
le Roux et al. (2007)^36^	Observational	UK	16	16	100	42 days	VAS (100 mm) 180 min	Mixed meal (1674 kJ)	‐	‐	‐	‐	‐	↑	‐
Laurenius et al. (2012)^37^	Observational	Sweden	43	42	98	6 weeks	VAS (100 mm) 60 min	Mixed meal (2354 kJ)	16%	42%	42%	‐	NS	↑	‐
Yousseif et al. (2014)^96^	Observational	UK	10	10	100	6 weeks	VAS(100 mm) 180 min	250 ml liquid (2092 kJ)	‐	‐	‐	↓	‐	↑	‐
Schmidt et al. (2016)^30^	Observational	Denmark	17	16	94	7 weeks	VAS (100 mm) 180 min	175 ml powder meal (1297 kJ)	41%	17%	42%	‐	↑	↑	NS
Bryant et al. (2013)^99^	Observational	UK	12	12	100	2 months	VAS (100 mm) 180 min	200 ml liquid (1255 kJ)	11%	0%	89%	NS	NS	↑	NS
Falken et al. (2011)^31^	Observational	Sweden	12	12	100	2 months	VAS (100 mm) 180 min	200 ml liquid (1255 kJ)	11%	0%	89%	↓	‐	↑	‐
Schmidt et al. (2016)^30^	Observational	Denmark	17	14	82	11 weeks	VAS (100 mm) 180 min	175 ml powder meal (1297 kJ)	41%	17%	42%	‐	↑	↑	NS
Borg et al. (2006)^101^	Observational	UK	6	6	100	3 months	VAS (100 mm) 180 min	Mixed meal (1757 kJ)	‐	‐	‐	↓	‐	↑	‐
Karakamos et al. (2008)^94^	Randomized controlled trial	Greece	12	12	100	3 months	VAS (100 mm) 120 min	Mixed Meal (1757 kJ)	16%	29%	55%	NS	NS	‐	‐
Svane et al. (2016)^61^	Observational	Denmark	9	9	100	3 months	VAS (100 mm) 300 min	Mixed meal (1260 kJ)	14%	33%	53%	↓	↑	‐	‐
Yousseif et al. (2014)^96^	Observational	UK	10	10	100	3 months	VAS (100 mm) 180 min	250 ml liquid (2092 kJ)	‐	‐	‐	NS	‐	↑	‐
Karakamos et al. (2008)^94^	Randomized controlled trial	Greece	12	12	100	6 months	VAS (100 mm) 120 min	Mixed Meal (1757 kJ)	16%	29%	55%	NS	NS	‐	‐
Borg et al. (2006)^101^	Observational	UK	6	6	100	6 months	VAS (100 mm) 180 min	Mixed meal (1757 kJ)	‐	‐	‐	↓	‐	↑	‐
Bryant et al. (2013)^99^	Observational	UK	12	12	100	12 months	VAS (100 mm) 180 min	200 ml liquid (1255 kJ)	11%	0%	89%	NS	NS	↑	NS
Cazzo et al. (2017)	Observational	Brazil	11	11	100	12 months	VAS (100 mm) 180 min	Mixed meal (2155 kJ)	17.5%	41.8%	40.7%	↓	↑	↑	↓
Falken et al. (2011)^31^	Observational	Sweden	12	12	100	12 months	VAS (100 mm) 180 min	200 ml liquid (1255 kJ)	11%	0%	89%	↓	‐	á	‐
Karakamos et al. (2008)^94^	Randomized controlled trial	Greece	12	12	100	12 months	VAS (100 mm) 120 min	Mixed meal (1757 kJ)	16%	29%	55%	NS	NS	‐	‐
Laurenius et al. (2012)^37^	Observational	Sweden	43	41	95	12 months	VAS (100 mm) 60 min	Mixed meal (4707 kJ)	16%	42%	42%	‐	NS	‐	‐
Pournaras et al. (2010)^102^	Cross‐sectional	UK	34	6	35	12 months	VAS (100 mm) 180 min	Mixed meal (1674 kJ)	10.2%	41%	48.8%	‐	↑	‐	‐
Pournaras et al. (2010)^102^	Cross‐sectional	UK	34	5	30	18 months	VAS (100 mm) 180 min	Mixed meal (1674 kJ)	10.2%	41%	48.8%	‐	↑	‐	‐
Schmidt et al. (2016)^30^	Observational	Denmark	17	21	53	18 months	VAS (100 mm) 180 min	175 ml powder meal (1297 kJ)	41%	17%	42%	‐	↑	↑	NS
Laurenius et al. (2012)^37^	Observational	Sweden	43	42	98	24 months	VAS (100 mm) 60 min	Mixed meal (4707 kJ)	16%	42%	42%	‐	NS	‐	‐
Pournaras et al. (2010)^102^	Cross‐sectional	UK	34	6	35	24 months	VAS (100 mm) 180 min	Mixed meal (1674 kJ)	10.2%	41%	48.8%	‐	↑	‐	‐
Thirlby et al. (2006)^65^	Observational	USA	76	43	57	32 months	VAS (1‐9) 180 min	Chocolate (Snickers) bar (1180 kJ)	5.7%	44.71%	49.68%	↑	↑	‐	‐

*Note*: All data measured as change from baseline following gastric bypass surgery. Data organized by follow‐up duration from shortest time postoperatively.

Abbreviations: NS, no significant change from preoperative values; VAS Visual Analogue Scale; ↓, significantly lower than preoperative values; ↑, significantly higher than postoperative values; −, data not reported.

Postprandial desire to eat was assessed in five studies, with no study reporting an increase in the postoperative phase. One study^64^ demonstrated a greater reduction in the desire to eat high‐ED foods (≥14.6 kJ/g) compared with low‐ED foods (<4.2 kJ/g). Similar changes were observed in postprandial hunger, with almost all studies reporting either a decrease or no change. One study^65^ observed an increase at 32 months postoperatively only, but a lack of long‐term follow‐up data means the significance of this finding cannot be established. Nine studies assessed postprandial fullness, with most reporting an increase post‐surgery.

**FIGURE 4:**
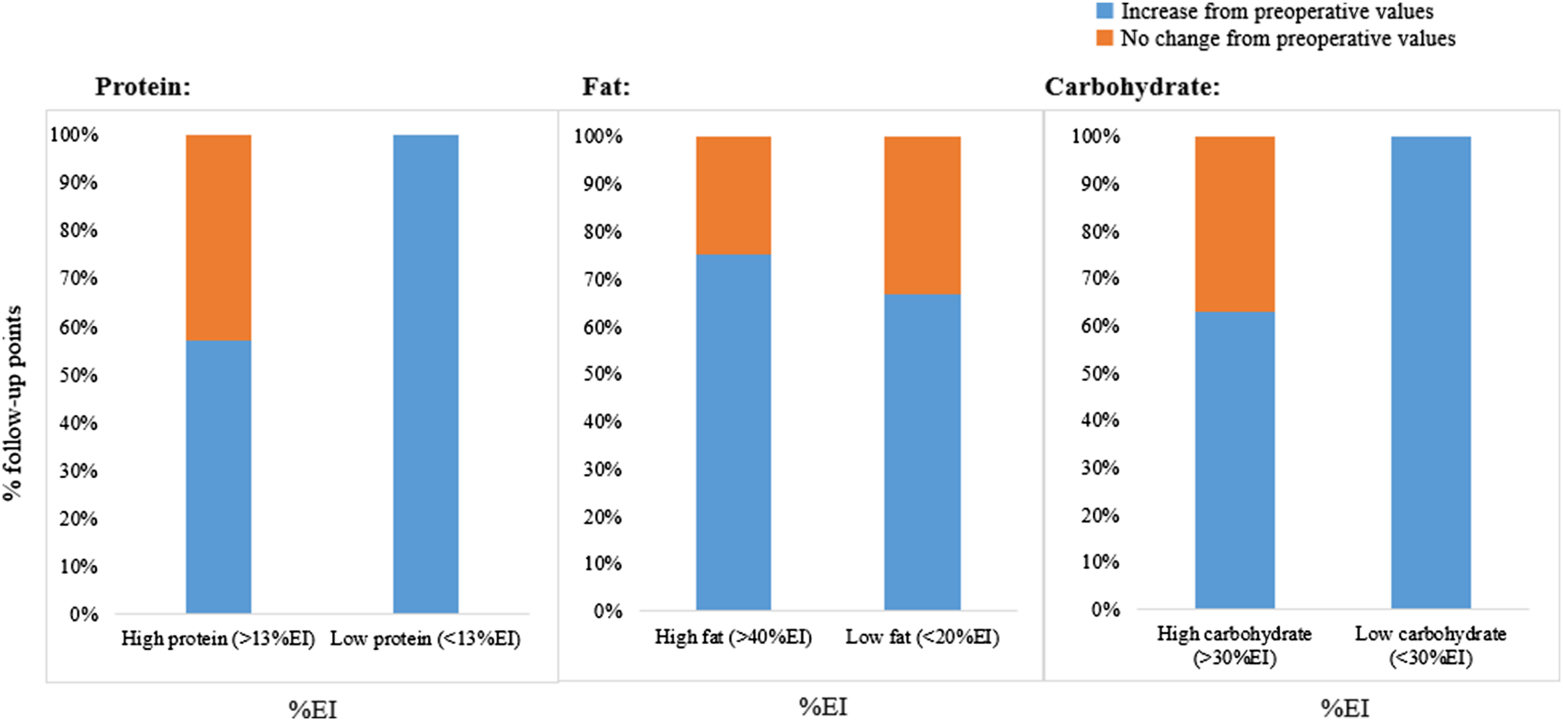
Percentage of follow‐up time points measuring changes in postprandial satiety from preoperative values in patients after surgery. Data grouped by relative macronutrient content of the test meal and sourced from papers summarized in Table 5

The format and formulation of the test meal given varied between studies. Either mixed meals (*n* = 6), liquid meals (*n* = 9), or snack foods (*n* = 2) were given to participants as a pre‐load, with energy content ranging from 1035 to 4707 kJ. Only one study adjusted meal size according to time postoperatively,^37^ and relative protein (6% to 41%), fat (0% to 45%), and carbohydrate (40% to 89%) composition of the meals showed large variation. To assess the possible influence of this variation on postprandial satiety, data were grouped by the macronutrient compositions and combinations proposed by Geiselman et al.^66^ Changes were grouped by protein (high [>13%] vs. low [<13%]) (Figure 4A), fat (high [>40%] vs. low [<20%]) (Figure 4B), and carbohydrate (high [>30%] vs. low [<30%]) (Figure 4C). All studies that employed low‐protein loads demonstrated an increase in postprandial satiety, whereas there was no definitive effect following a high‐protein load. There were also no clear differences between the administration of a high‐ versus low‐fat or high‐ versus low‐carbohydrate load. The influence of the test meal is an important consideration when interpreting these data, but more information is required in bariatric patient groups.

### 3.7 Objectively measured appetite

Three studies used direct measures of EI to objectively measure appetite^37^, ^61^, ^62^ and all three reported a decrease in appetite from pre‐ to post‐surgery. Postoperatively, objective appetite decreased from pre‐surgery values in two studies,^61^, ^62^ whereas Laurenius et al.^37^ observed a decrease in overall meal size and eating rate (g/min). These studies measured subjective appetite in conjunction with objective appetite, but postoperative changes in subjectively assessed fasted and postprandial appetite did not always correspond with the decrease in objectively measured ad libitum EI.

Biochemical assessments were made in 15 studies, with PYY (*n* = 11), GLP‐1 (*n* = 10), and ghrelin (*n* = 7) measured most frequently. The majority of studies observed no change from pre‐surgery in fasting levels of PYY, GLP‐1, or cholecystokinin (CCK), whereas most reported a decrease in ghrelin, insulin, and leptin. Postprandial appetite hormone responses included an increase in CCK, PYY, and GLP‐1. Cazzo et al.^67^ also followed up postprandial GLP‐2 and observed an increase from pre‐ to post‐surgery. Most studies reported a decrease in postprandial ghrelin and leptin, whereas reported changes in postprandial insulin were inconsistent.

Finally, it is worth noting the inconsistencies in the method of presenting both biochemical and subjective results. Most frequently, findings were presented as area under the curve (AUC) but were also presented as mean values or as intrameal AUC. Furthermore, time point 0 was not standardized across studies, and the 0‐min measurement could have been before or after consumption of the test meal or may not have been included when presenting AUC.

## 4 DISCUSSION

The overall findings from this systematic review demonstrated that only a decrease in total EI and an increase in postprandial satiety remain different from preoperative values for more than 6‐month post‐surgery, whereas relative macronutrient intake and premeal hunger remain unchanged. Given the paucity of follow‐up data on patients for longer than 3 years, conclusions cannot currently be drawn on the impact of these changes on the longer term weight trajectory following gastric bypass surgery.

As with other systematic reviews evaluating outcomes following gastric bypass surgery, the findings of this review are constrained by the quality and consistency of the available evidence.^39^, ^40^, ^41^ Most notably, an overreliance on subjective methodology, the lack of standardization in the measurement and presentation of data, and the shortage of longer term studies (>3 years) make it difficult to draw meaningful conclusions from the available data.

### 4.1 Measurement of dietary intake

All studies used subjective self‐reported measures to assess EI. These measures are based on the tacit assumption that data obtained by these methods are a valid representation of habitual intake. However, misreporting of EI, specifically underreporting, is a limitation of these methods and is particularly prevalent in individuals with obesity.^38^ A major concern is the failure to acknowledge the phenomenon of misreporting of food intake in the bariatric surgery literature. Only three studies documented misreporting,^37^, ^49^, ^50^ but they were inconsistent in both their calculation and interpretation of misreporting.

To date, there has been only one research group that has used objective measures in any bariatric population. Following administration of an ad libitum buffet meal that consisted of 20 items varying in both fat content (high vs. low) and taste (sweet vs. savory), they observed no significant changes in relative macronutrient contribution or food selection from pre‐ to post‐surgery, although there was a significant postoperative reduction in EI. Participants were followed up at 6^68^ and 18 months,^69^ and findings support the conclusions of this review that macronutrient contribution to EI remains consistent relative to baseline beyond 6‐month post‐surgery.

The reasons why individuals with obesity are more likely to underreport are not well understood, but it probably represents an interplay between cognitive and behavioral processes.^70^, ^71^ Underreporting can be conscious or subconscious and may be affected by negative social attitudes toward carrying excess weight and subsequent guilt about the quantity or type of food consumed.^72^ Macronutrient‐specific underreporting is difficult to assess as relative macronutrient intake is highly interrelated, but it is conceivable that foods and macronutrients with perceived negative health connotations (e.g., high‐fat, high‐sugar foods) are underreported, whereas those with perceived positive attributes (e.g., high‐protein foods, fruits, and vegetables) are overrepresented. However, at present, there is no way of verifying whether EI underreporting is also associated with macronutrient‐specific misreporting.

Available methods for calculating misreporting were developed for weight‐stable populations.^73^, ^74^, ^75^ Whereas previous work has attempted to assess the efficacy of these methods in preoperative bariatric surgery candidates,^76^ no studies to date have established their efficacy and limitations in assessing EI postoperatively. In the absence of objective, independent validation techniques for assessing the validity of EI data, some studies have attempted to assess the relative validation of their data by comparing the findings of one subjective measure with findings from another subjective method of dietary assessment. However, given that all subjective methods of dietary assessment are susceptible to the same inherent and intrinsic errors, this does not provide a measure of absolute validity.^38^ The inability to measure accurately dietary intake in free‐living individuals is an intractable problem across nutrition research,^77^ and further work is urgently required to establish the magnitude and direction of reporting bias in gastric bypass patients, particularly if there is a continued reliance on subjective self‐reported measures of dietary intake. Until then, the validity of self‐reported EI by gastric bypass patients should be treated with considerable caution.

Another challenge when using subjective methods of dietary assessment is the inability to measure portion size accurately. The difficulties that face all individuals when documenting portion size are known,^78^ and a significant reduction in postoperative portion sizes^79^ means that methods that rely on predefined portion sizes (e.g., artificial food models) may not accurately represent reduced portion sizes post‐surgery. Less than half of studies specified the use of a tool to improve the accuracy of portion size reporting, and although there is little research evaluating the efficacy of these tools in the bariatric surgery context, the methods employed should be flexible enough to accommodate fluctuating portion sizes.

A major focus of this review was to evaluate changes in relative macronutrient intakes as these may be more indicative of changes in food preferences, which may influence long‐term weight loss and maintenance. Although some studies have also presented macronutrient data in absolute (g/day) values,^12^ ideally, data should be presented as both absolute and relative values to aid the interpretation of findings.

Inconsistencies in the presentation of results were also evident in the measurement of food selection. In the limited studies that measured these changes, food groups were researcher defined, and both the definition and reporting of these changes were highly variable. Intake was reported as either servings/day, %EI, or %patients classified as “frequent” (>4 days a week) consumers.

Finally, when sugar intakes were reported, the definition used was not consistent or comparable between studies. Only three studies measured sugar intake independently of total carbohydrate,^50^, ^51^, ^52^ and by not considering sugar intake separately to carbohydrate, important dietary shifts may have been obscured. For example, Kenler et al.^55^ reported a significant decrease in sugar‐sweetened beverage intake that had no corresponding impact on relative total carbohydrate intake but did not report changes in relative sugar intake which may have been present.

### 4.2 Measurement of appetite

Although the subjective assessment of appetite using VASs may, in theory, be easy to administer, their limitations need to be acknowledged, including difficulties in quantification as scores are not representative of absolute values.^80^ Most studies reviewed combined subjective measures with either biochemical assessments or independently observed EI, but these objective measurements did not always correspond with subjectively reported changes suggesting that VAS may not be sensitive to changes in appetite sensations in post‐surgery patients.

When measuring premeal hunger sensations, inconsistencies in the measurement protocols make cross‐study comparisons difficult. The duration of fast prior to measurement ranged from 5 to 12 h, with one study not stipulating a period of fasting at all.^63^ Antecedent diet was also not standardized or recorded by any study, despite previous work in nonsurgical populations that observed a significant difference in the measurement of premeal hunger following consumption of different antecedent diets.^81^ Physiological changes following gastric bypass surgery may further exaggerate the effect antecedent diet may have on premeal hunger, but this effect has yet to be evaluated.

In the measurement of postprandial appetite sensations, there were notable inconsistencies between studies in the format and formulation of the test meal presented. The effect of different test meals on both objectively and subjectively measured appetite has not been well documented in patients after bariatric surgery, but in nonsurgical populations, meal structure has been observed as impacting on subjectively assessed appetite.^82^ The texture and formulation of test meals offered have also been observed to influence appetite hormone responses,^83^ which may be of particular significance in the bariatric surgery context.

Across studies, there were differences in the definition of postprandial appetite and the descriptive terminology applied. Although postprandial satiety was the most frequent measure of postprandial appetite, the term “satiety” was used interchangeably with terms for other appetite sensations (hunger, fullness, and desire to eat). The term “satiety” refers to the state in which the initiation of further eating is suppressed after the completion of a meal and the substitution of other appetite sensations invalidates conclusions. In contrast, satiation is a process that occurs during eating that brings eating to a stop, and the inconsistent reporting of “0 min” (before or after completion of test meal) and the sporadic inclusion of “0 min” in AUC calculations of postprandial appetite make comparisons difficult. It could be argued that the “0‐min” measurement is affected by sensory cues, whereas subsequent measurements reflect post‐ingestive cues and so should be presented independently of each other. Many of the studies in this review failed to fully distinguish between satiety and satiation. Although the use of different terminologies may ultimately describe a similar functional outcome, studies should be explicit as to what is being measured to avoid possible misinterpretation of outcomes.

### 4.3 Other methodological considerations

Other methodological issues that may influence the outcome of studies assessing food intake in bariatric patients include participant recruitment and duration of follow‐up. Due to difficulties in the recruitment and retention of patients after gastric bypass, most studies recruit at one site where patients are accessible. Although this is entirely logical, it would be beneficial if studies included relevant information about the postoperative care and dietary guidance patients receive to further inform the interpretation of findings. Current dietary advice given to patients is not uniform, and so it is conceivable that differences in the information received may affect patient behaviors and, in turn, results.

Participant retention in studies was highly variable, with some studies reporting a completion rate of less than 30%. Lack of adherence to clinical follow‐up is associated with poorer outcomes,^84^ and although attrition of patients after gastric bypass has not been evaluated in the context of dietary intake or appetite measurements, it could be surmised that those who drop out are not adhering to postoperative recommendations. Those who are lost to follow‐up may not experience comparable postoperative changes, and so findings may be obscured or exaggerated. There is a lack of transparency in the documentation of reasons for participant dropout.^85^ As most studies in this review were observational, there are strong arguments that the development of study protocol guidelines specifically for bariatric research (similar to what exists for RCTs^86^ and observational studies^87^) would enhance reporting and ensure comparability of results.

The disproportionate representation of studies with short‐term follow‐up is highly likely to be contributing to inconsistencies in the current literature. Patients progress through diet stages (liquid, pureed, soft) in the first 3‐month post‐surgery,^88^ and typically postoperative weight loss is initially large before plateauing at 18–24 months' post‐surgery.^89^ The assessment of appetite in particular is mostly carried out only up until 6‐month post‐surgery and is most unlikely to provide an informed evaluation of the impact on longer term body weight regulation.

## 5 CONCLUSION

In conclusion, after gastric bypass surgery, patients have a profound decrease in EI, which appears to be accompanied by an increase in postprandial satiety. Moreover, these postoperative consequences appear to be robust and maintained up to 3 years after gastric bypass surgery. However, only tentative conclusions can be drawn from the available literature as there is a paucity of studies with objectively validated assessment of changes in dietary intake and appetite in patients after gastric bypass in the medium and long term. Inconsistencies in the methods, analysis, interpretation, and presentation of results are highly likely to be contributing to the confusion and remain major obstacles in bariatric research. The critical gaps in understanding the dynamics of food selection and intake following bariatric surgery will only be filled by the application of fit‐for‐purpose methodology and by reaching a consensus on reporting criteria.

## CONFLICT OF INTEREST

The authors have no conflict of interest.
